# Harmonic enhancement to optimize EOG based ocular activity decoding: A hybrid approach with harmonic source separation and EEMD

**DOI:** 10.1016/j.heliyon.2024.e35242

**Published:** 2024-07-29

**Authors:** Çağatay Demirel, Livia Reguş, Hatice Köse

**Affiliations:** a Computer Engineering Department, Istanbul Technical University, Maslak, 34467 Sarıyer, Istanbul, Turkey; b Donders Institute for Brain, Cognition and Behaviour, Kapittelweg 29, Nijmegen, 6525 EN, Netherlands; c Department of Social, Health and Organizational Psychology, Utrecht University, Utrecht, Heidelberglaan 1, 3584 CS, Netherlands; d AI and Data Engineering, Istanbul Technical University, Maslak, 34467 Sarıyer, Istanbul, Turkey

**Keywords:** Electrooculogram, Signal processing, Harmonic source separation, Ensemble empirical mode decomposition, Hilbert-Huang transform, Deep learning, Harmonic ratio

## Abstract

Intelligent robotic systems for patients with motor impairments have gained significant interest over the past few years. Various sensor types and human-machine interface (HMI) methods have been developed; however, most research in this area has focused on eye-blink-based binary control with minimal electrode placements. This approach restricts the complexity of HMI systems and does not consider the potential of multiple-activity decoding via static ocular activities. These activities pose a decoding challenge due to non-oscillatory noise components, such as muscle tremors or fatigue. To address this issue, a hybrid preprocessing methodology is proposed that combines harmonic source separation and ensemble empirical mode decomposition in the time-frequency domain to remove percussive and non-oscillatory components of static ocular movements. High-frequency components are included in the harmonic enhancement process. Next, a machine learning model with dual input of time-frequency images and a vectorized feature set of consecutive time windows is employed, leading to a 3.8% increase in performance as compared to without harmonic enhancement in leave-one-session-out cross-validation (LOSO). Additionally, a high correlation is found between the harmonic ratios of the static activities in the Hilbert-Huang frequency spectrum and LOSO performances. This finding highlights the potential of leveraging the harmonic characteristics of the activities as a discriminating factor in machine learning-based classification of EOG-based ocular activities, thus providing a new aspect of activity enrichment with minimal performance loss for future HMI systems.

## 1 Introduction

Due to an aging population and the increase in chronic health problems worldwide, global reports on disabilities have raised concerns. Technological advancement has attempted to overcome these health problems, including motor disabilities, through intelligent robotic systems [1]. Moreover, there has been an ascent in developing interactive assistive devices, which has accelerated in recent years. These include automated wheelchairs and verbally and posturally controlled mechanics [2], [3], [4]. However, people with locked-in syndrome (LIS), a rare neurological illness resulting in partial or complete stroke [5] due to injured parts of the brainstem, are limited to eye movements only. Additionally, some patients with neurodegenerative diseases, namely amyotrophic lateral sclerosis (ALS) or multiple sclerosis (MS), are unable to operate these devices [6]. Consequently, those suffering from such diseases require non-invasive human-machine interaction (HMI)-based assistance as an alternative way of regaining their mobility [7].

HMI systems predominantly comprise two possible inputs: electroencephalogram (EEG) or electrooculogram (EOG). There are numerous benefits to using EOG over EEG-based HMI pipelines, including significantly less expensive and lightweight equipment, higher temporal resolution, and smaller data streams with a higher discriminant factor for detecting an ocular event. It has been shown that single-channel EOG devices could achieve the same goal as bulky EEG options to yield various HMI systems [7], [8]. Higher precision around the ocular muscles gives better information to represent the action. Moreover, it decreases the need for artifact reduction [9], considering that EOG activity itself could be considered an artifact voluntarily created by the user.

Further, most studies developing EOG-HMI-based systems have developed decision-making models based on the blink and saccadic potentials [10]. As a result of these findings relying on EOG time-domain potential oriented rule-based systems, complex ocular activity recognition hasn't been developed using a single-channel EOG signal.

The literature suggests that in the past few years, various multi-channel designs that are not practical but can provide acceptable real-time performance have been developed. For instance, using Ag-AgCl surface EMG electrodes, four different activities (right, left, up, and down) are estimated from a five-channel system [11]. In another study, five eye movements are estimated using six Ag-AgCl EMG electrodes [12]. Some studies gauge only eye blinks with a single-channel EOG [13], [14]. The conclusion drawn from these studies is that minimal-sensing a single-channel EOG electrode in conjunction with a reference channel EOG devices should be avoided to provide comprehensive control. These either employ saline-based multiple EMG surface electrodes or rule-based control using simple eye-blink artifacts for better referencing across eyes [15], a time-consuming and cumbersome setup circumventing the goal of an effortless solution for impaired patients. Given that they have limited capabilities and skills for device placement, a single-channel EOG-based HMI approach would be a logical option to follow [16], [17], despite the fact that this technique also brings some challenges in the signal analysis step.

In this vein, there are recent studies on EOG-based activity decoding which provide various real-world solutions. One study develops an asynchronous EOG speller system for efficient communication using single blinks or winks, aiding individuals with motor disabilities in selecting characters accurately [18]. Another study creates an EOG system for controlling a serious game using eye movements, enhancing interaction for people with motor disabilities [19]. Research on closed-eye gaze gesture passwords with EOG sensors in smart glasses improves mobile authentication security [20]. Additionally, detecting driver sleep onset using slow eye movements and a bimodal LSTM network enhances safety [21]. Another study proposes a pipeline combining statistical and deep learning features to detect fine-grained reading activities from an EOG dataset collected in real-world settings providing a significant performance on reading activity detection [22]. These advancements demonstrate how optimizing EOG decoding performance can further provide a more parsimonious way of addressing real-world problems and enhance potential applications, making ongoing research in this field crucial for future technological developments.

A significant challenge in analyzing biopotential signals, such as those from EOG, is the inherent non-stationarity of these signals. Non-stationary signals pose several challenges in decoding as they change their statistical structure (e.g., mean, variance) over time [23]. These signal types can stem from such factors as muscle tremors, short- and long-term fatigue, and the non-stationary nature of muscle stress. Experimental results reported in a study showed that the decoding accuracy of the classification model decreased significantly considering muscle fatigue, forearm angle, and acquisition time, respectively [24]. Additionally, another study [25] found that the mean power frequency decreased as a linear function of fatigue time, indicating the lead distortion of harmonic composition. Saccadic artifacts could also arise during prolonged static activities, such as looking at a screen for a long time [26]. These findings formalize the dynamic variations during regular staring activity, which is a static activity. Moreover, they suggest that non-stationary signals are challenging to decode due to various factors and decoding accuracy may decrease over time, making it necessary to employ appropriate preprocessing techniques.

Dynamic noise factors, particularly in some frequency components of EOG signals tend to accumulate at higher frequencies. Low-pass filtering is a common preprocessing step to remove such artifacts from EOG signals. For example, several studies [27], [28], [29] used low-pass filtering between 20 Hz to 35 Hz. However, the high-frequency range, which forms a part of the harmonic scale and may include complementary components essential for defining specific ocular activities, risks being lost if low-pass filtering with a low threshold within the Nyquist scale is applied, potentially eliminating significant information. Accordingly, the study [30] reported that after low-pass filtering, depending on the filter's cutoff frequency, the information flow regarding the transfer of entropy was significantly misled, leading to a damping of the accuracy of the entropy. Furthermore, [31] found that preprocessing without a solid prior on the to-be eliminated artifact disrupts the data information content and time order, leading to spurious and missed causalities. These findings suggest that while low-pass filtering can effectively remove high-frequency noise and artifacts, setting the threshold considerably low may also lead to incomplete signal representation and the loss of vital information. Alternative methods should be explored to clean non-oscillatory components across the frequency spectrum and mitigate trade-offs.

**Figure 1:**
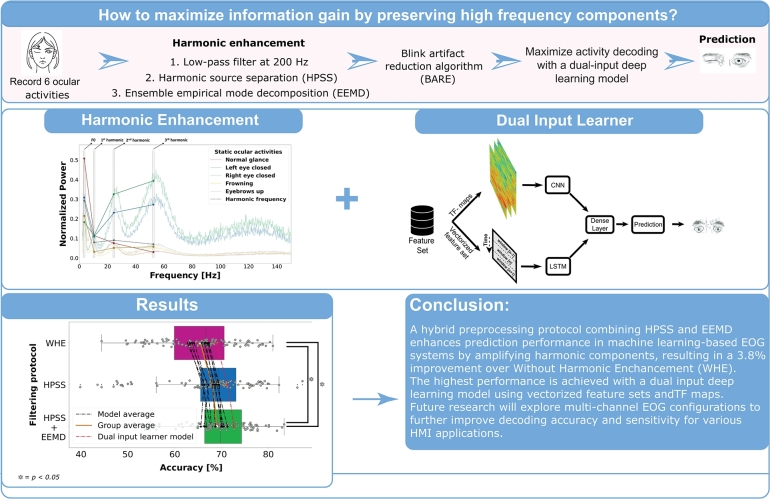
Schematic overview of the study.

In this study, by preserving the high-frequency content of the EOG data, an alternative hybrid preprocessing technique combining harmonic source separation and ensemble empirical mode decomposition is developed to boost ocular activity decoding. The preprocessed data, amplifying the harmonic components, is then utilized in multiple machine learning scenarios. A dual-input deep learning model is developed to combine spectrogram images and vectorized scalar feature sets of consecutive overlapping windows, which surpassed the other selected classifiers. Subsequently, the classification performances of static ocular activities from the best-performing model are investigated by correlating these with the harmonic ratios of their averaged Hilbert-Huang spectra. Accordingly, the effectiveness of the harmonic intensities of the eye movement activities is highlighted based on their classification performances. The visual depiction of the study overview is shown in Fig. 1.

## 2 Experiments & methods

### 2.1 Overview of the EOG activity decoding protocol

**Figure 2:**
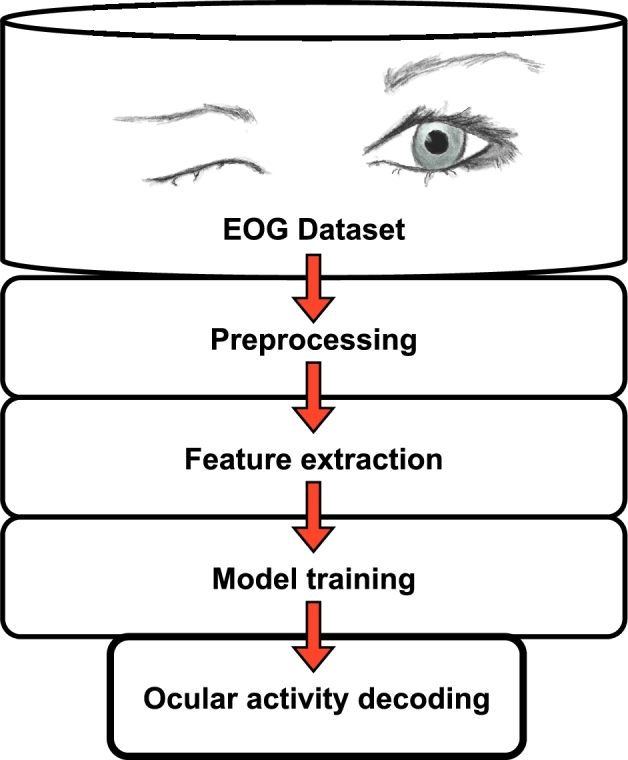
Overall flow diagram of EOG activity decoding pipeline.

Each subject is initially instructed to carry out all visual functions individually. The preprocessed EOG signals undergo feature extraction, feature elimination, and low discriminative feature removal. The feature set is trained using several machine learning models and placed to the test in the final step. The overall flow diagram of the EOG-based activity decoding pipeline is shown in Fig. 2.

### 2.2 Dataset collection

EOG data is collected using the MindWave Headset from Neurosky (NeuroSky, San Jose, CA, USA) on nine healthy participants, including six males and three females (the age range is between 23 to 30 (mean: 26.8, std: 1.9)). The device is a wireless EOG headset run on Bluetooth connection with a single-dry electrode referenced with an additional ear electrode.

**Figure 3:**
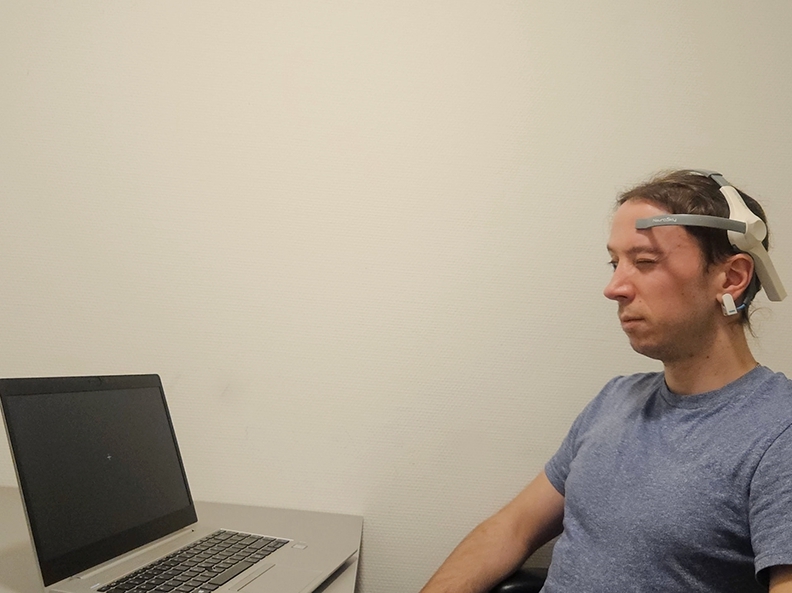
Example of the experimental setup featuring the left eye closed activity.

**Figure 4:**
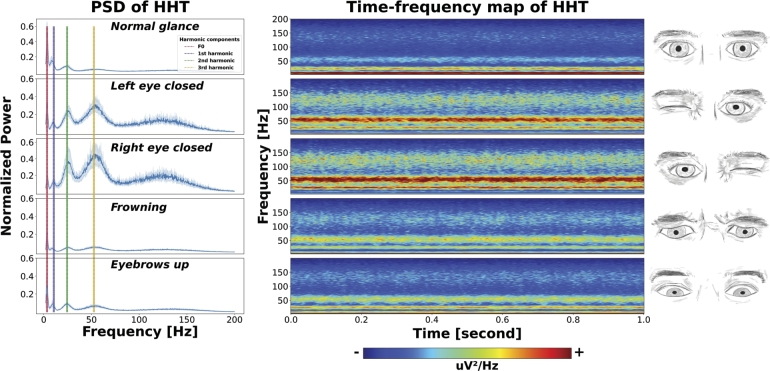
Ocular activities with their corresponding HHT time and frequency distributions. The left panel displays the normalized power spectral density (PSD) distribution obtained from HHT for each activity. The fundamental frequency (F0) is detectable around 3.5 Hz, with three harmonics identified at 10.5 Hz, 24.5 Hz, and 52.5 Hz. Although the frequencies of all activities are similar, the distinctive characteristics of each activity vary through the differences in energy distribution across frequencies. The average time-frequency HHT spectrograms are depicted in the middle panel. Notably, the energy levels of the harmonic frequencies remain static in the time domain in lower frequencies. The normal glance activity exhibits the lowest harmonic energy, while frowning and eyebrows-up activities show minimal power, except around 52.5 Hz. These observations suggest that the IMFs, especially those responsible for high frequencies, do not possess sufficiently prominent energy. This outcome is explained by the significant energy drop observed in all activities above the 3rd harmonic at 52.5 Hz. This can give the impression that there are no more prominent harmonic peaks. Remarkably, frowning activity, which induces muscle tremor more than any other activity, does not exhibit the expected power fluctuations in high frequencies. This outcome may be explained by the fact that these are percussive components, reduced during the harmonic enhancement process. However, the total energies of the left and right eye closed activities are substantially higher than those of other activities and exhibit fluctuating activity between 100 Hz and 150 Hz. In the rightmost panel, the five actions are presented from top to bottom: normal glance, left eye closed, right eye closed, frowning, and eyebrows up.

The subjects performed five different static ocular activities, including left eye closed, right eye closed, frowning face, eyebrows up, normal glance, and constant eye blinking as a dynamic activity. The participants executed the ocular activities for 60 seconds, including a 20-second practice period and a 40-second experimental period. Throughout the 60-second duration, they engaged in eye movements while gazing at the eye fixation cross located in the center of the monitor. EOG data are acquired from each subject in a single session for all six activities, representing a total experimental time of 240 seconds. Fig. 3 depicts an example of the experimental setup, with the consent for using the image. Additionally, Fig. 4 gives a comprehensive overview of the chosen activities.

The EOG device used in this study has a sampling rate (Fs) of 500 Hz and a 16-bit depth. Post recording, the raw data are divided into 1-second windows with padding of 500 ms. Each window is labeled with the corresponding eye action before inclusion in the dataset.

### 2.3 Robust z-score normalization of a raw signal

Initially, a 50 Hz notch filter (IIR) with a band-pass filter between 2 to 200 Hz is applied to the raw EOG signals to hinder the artifacts. These artifacts arise with sweat and are irrelevant for ocular or neural activities [32]. Next, EOG time-series data are standardized and normalized using robust z-score normalization. This step solves the difficulties brought on by artifacts and asymmetry in EOG signals. A sturdy measure of central tendency and dispersion is produced via robust z-score normalization, subtracting the median and dividing the result by the median absolute deviation (MAD) [33]. Outliers and non-Gaussian distributions are effectively diminished with this method, leading to a standardized scale for the EOG time-series data. This normalization approach ensures consistent and comparable data between subjects. Additionally, the multiplication of MAD by 0.6745 in the variation of robust z-score normalization serves as a correction factor, ensuring consistency with the standard deviation of a normally distributed dataset. The formula of robust z-score normalization is shown in Eq. (1).

(1) MAD=median(|S−median(S)|)R(S)=0.6745x(S−median(S))/MAD

where R(S) is a robust z-score normalization of a given time-serie *S*.

### 2.4 Harmonic / percussive source separation

**Figure 5:**
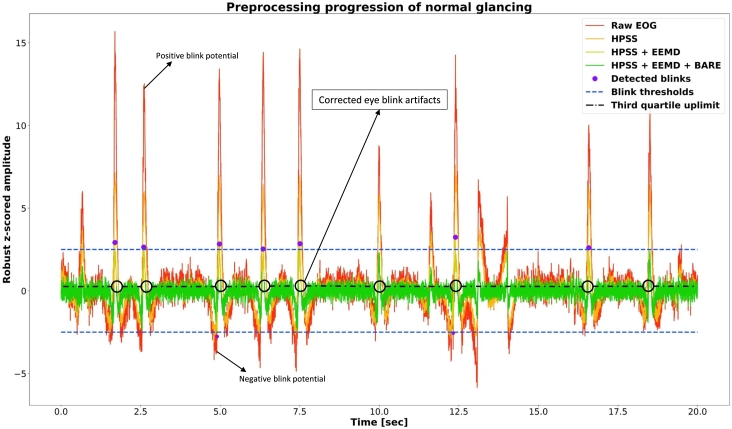
Comparison of harmonic filter and blink artifact correction algorithm on the time-domain signal. After a preliminary preprocessing stage, the HPSS filter cleans the signal of percussive elements by removing blink artifacts. All points above the peak and the top of the third quartile of the narrow fraction surroundings are replaced with the same indices in the envelope of the same signal using the novel blink artifact correction technique.

In this study, the goal is to be able to operate high-frequency components reflected from F0, as the harmonics composition of the eye movements, including those in the higher frequency range, can provide valuable insights into the eye movement characteristics. The peaks that are exact multiples of each other in the spectral domain are identified as the harmonic representation of a given activity. Next, the harmonic/percussive source separation (HPSS) algorithm [34] isolates the harmonic and percussive components. The first step in this process involves transforming the data into the spectral domain via the short-time Fourier transform (STFT) [35], setting the stage for the HPSS algorithm to operate. Percussive components do not follow the harmonic pattern and act as the noise of a time series. Utilizing the anisotropic smoothness of power spectrograms, or the fact that harmonic power spectrograms are continuous in the time direction and percussion power spectrograms in the frequency direction, is one of the keys to HPSS. Harmonic events are highlighted in a given EOG signal by removing continuous noisy sequences in the frequency domain associated with percussive components. The design of the algorithm, based on a median filter, also plays a role in eliminating signal drift. After using HPSS, the time-domain signal is obtained by reconstructing the spectral domain signal using an inverse short-time Fourier transform (ISTFT). Eq. (2) depicts the formula of the harmonic filter. Examples of raw, percussive, and harmonic-filtered EOG signals are shown in Fig. 5.

(2) $$H_{i}=M(S_{h},\;l_{\left(\mathrm{harm}\right)})$$

where *M* is a median filter, $S_{h}$ is a median filtering frequency piece, $l_{\left(harm\right)}$ is the length of the harmonic median filter, and $H_{i}$ is the harmonic filtered signal in frequency spectrum. Median filters transform the given signal piece into the median of the signal values (see Eq. (3)).

(3) y(n)=median(x(n−k:n+k),k=(l−1)/2)

The input vector is given as x(n), and the output y(n) is a median filtered signal of length *l*. Here *l* represents the number of samples for which the median filtering algorithm is implemented, with *l* representing an odd number.

### 2.5 Ensemble empirical mode decomposition

This section briefly reviews the original empirical mode decomposition method (EMD), which allows the decomposition of complex data sets into a finite number of ‘intrinsic mode functions’ (IMFs). The IMFs are simple oscillatory functions with varying frequency and amplitude and admitting well-behaved Hilbert transforms [36]. The EMD method consists of extracting oscillatory modes in the signal through the sifting process, which separates the signal into its different frequency components (IMFs). For instance, using EMD decomposes the data as in Eq. (4).

(4) $$x(t)=\sum_{j=1}^{n}{\mathrm{imf}}_{j}+r_{n},$$

where x(t) is the recorded data containing the signal and noise, $r_{n}$ is the residual of x(t), after the extraction of *n* number of IMFs, $imf_{j}$ [37]. EMD (combined with Hilbert spectral analysis [38]) has significant advantages for non-linear and non-stationary data analysis. It effectively utilizes the data, defines the local frequency without relying on the entire signal, and represents the first local and adaptive method in frequency-time analysis [36]. However, there is a major drawback to the original EMD method consisting of a single IMF containing signals of widely disparate scales or a signal of the same scale being present in various IMF components, leading to unreliable results - termed mode-mixing.

Ensemble empirical mode decomposition (EEMD) is a signal processing method that decomposes a signal into its IMFs by adding white noise to the original signal and then applying EMD to overcome the drawbacks of the latter [37]. By performing EMD multiple times with added white noise, EEMD generates multiple realizations of IMFs, ensuring more robust and reliable decomposition. The final result is obtained by averaging these realizations, resulting in reduced mode-mixing and increased accuracy in the signal decomposition. EEMD effectively processes non-linear and non-stationary signals, making it a valuable tool in various fields, including biomedical signal processing [39], [40]. The EEMD formula is given in Eq. (5).

(5) $$\text{EEMD}(x(t))=\frac{1}{N}\sum_{i=1}^{N}\text{EMD}(x(t)+w_{i}(t))$$

where x(t) represents the original signal, $w_{i}(t)$ is the white noise added in the *i*-th ensemble, *N* is the number of ensembles, and the average of the decompositions over *N* ensembles gives the EEMD signal x(t).

Both EMD and Complete Ensemble Empirical Mode Decomposition with Adaptive Noise (CEEMDAN) [41] are explored as variations of EEMD. However, EEMD proved to be the most effective approach, surpassing the other variations in performance. This superiority of EEMD justifies its selection for the extraction of IMFs.

Besides, given that the extremities in both tails (1^st^ and last IMFs) could have tendencies to catch artifactual components in either direction (baseline drifts and/or high-frequency disturbances), three variations of IMF summations are examined: removing the 1^st^ IMF, last IMF and merging all IMFs to restructure the time-series. The prediction performance is the highest when utilizing the entire IMF range, thus no IMF pruning is chosen.

The EEMD calculation is performed with the following parameters: the maximum number of IMFs set to 5, the number of ensembles set to 96, the ensemble noise level set to 1, and a threshold for stopping sifting at 0.05. The entire collection of IMFs is summed up as a reconstruction of the time-domain signal.

### 2.6 Conceptual framework of combining HPSS and EEMD as a hybrid approach

HPSS filters out percussive and transient noise elements, leaving a signal enriched with harmonic components. EEMD further amplifies these properties through an adaptive decomposition process. By focusing selectively on the harmonic aspects of the already filtered signal, EEMD isolates IMFs that encapsulate the signal's frequency scales. The rationale behind combining the above-described HPSS and EEMD is to refine and highlight the subtle harmonic nuances critical to emphasize the signal's harmonic structure. Within the scope of the proposed study, this step is referred to as *harmonic enhancement*. The noise-assisted nature of EEMD, which incorporates added white noise to prevent mode mixing [37], aims to work synergistically with HPSS's noise reduction to produce a set of IMFs representing the more approximated harmonic behavior of the signal. This decomposition allows EEMD to isolate the quick, transient movements (such as blinks or micro-movements) from the more stable components associated with the static ocular activity. These artefactual micro-ocular movements lack explicit characteristics, rendering the patterns to overshadow the harmonics. By decomposing the signal into IMFs, EEMD aims to segregate components associated with non-characteristic signals from those related to EOG activities as a way of harmonic extraction [36][42].

By preserving high-frequency components up to 200 Hz, this integrated approach enhances the distinct characteristics of specific EOG activities within the high-frequency spectrum, recognizing that the harmonic properties retained at higher frequencies act as reflections of the fundamental frequency. This method effectively distinguishes potentially relevant information from high-frequency noise and artifacts, thus circumventing the loss of information commonly incurred through intensive low-pass filtering.

### 2.7 Envelope insertion based blink artifact correction algorithm

Blinking represents a biological process consisting of the rapid closure of the eyelid in a semi-autonomous manner [43]. It can occur spontaneously as a reflex response, without conscious effort resulting from activity in the premotor brainstem. Blinking can also happen when triggered by external stimuli yet the movement is faster. Comparative studies suggest conflicting results when comparing spontaneous and corneal reflexive blinking. This includes finding the mean down phase range of voluntary blink activity significantly higher than reflexive blinking [44], and that there is no difference between these activities [45].

Blinking is unavoidable while recording eye-movement activity, yet it is crucial to distinguish between voluntary and involuntary actions for developing an HMI system. With this in mind, a novel blinking artifact removal (BARE) algorithm is developed. First, the recorded eye-movement data are passed through the basic preprocessing and harmonic filtering steps described above. The subsequent step is to correct the artifacts due to involuntary blinking. Since each EOG data is robust z-score normalized, the amplitude levels are practically at a comparable magnitude for all subjects and all ocular activities. However, post-visual inspection, a lower limit is determined to detect blink artifacts. Peaks above this limit are tagged as blink artifacts if they are at a certain distance from each other. The envelope insertion-based BARE method is presented in Algorithm 1. The positive and negative peaks (p_ind, p_ind_neg) are determined based on the lower ($q_{1}$) and upper quartile ($q_{3}$) limits of the whole signal. The area within these two boundaries is defined as the interquartile range and determines the inlier region of the EOG data during a repetitive task. In the subsequent step, the Savitsky-Golay filter of the absolute value from the signal is taken to extract the envelope.

**Algorithm 1:**
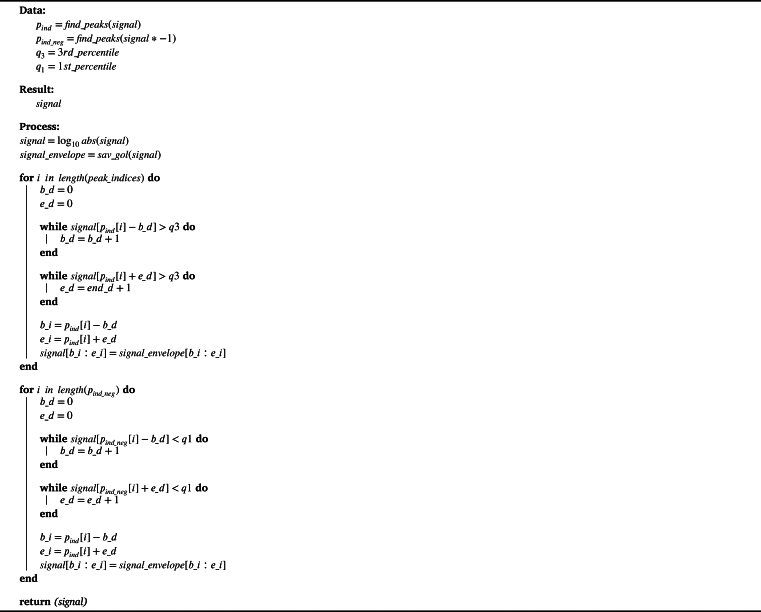
Envelope insertion based blink artifact correction algorithm (BARE).

Next, the fraction of narrow band parts on the right and left sides of the determined peak are chosen between the $q_{3}$ limit in the positive direction and the $q_{1}$ limit in the negative extreme. This region determines the area where the artifact is effective. The enveloped part of each detected region replaces the artifact within a loop. This way, artifact gaps in the EOG time series are reconstructed without losing the activity-related signal information.

**Figure 6:**
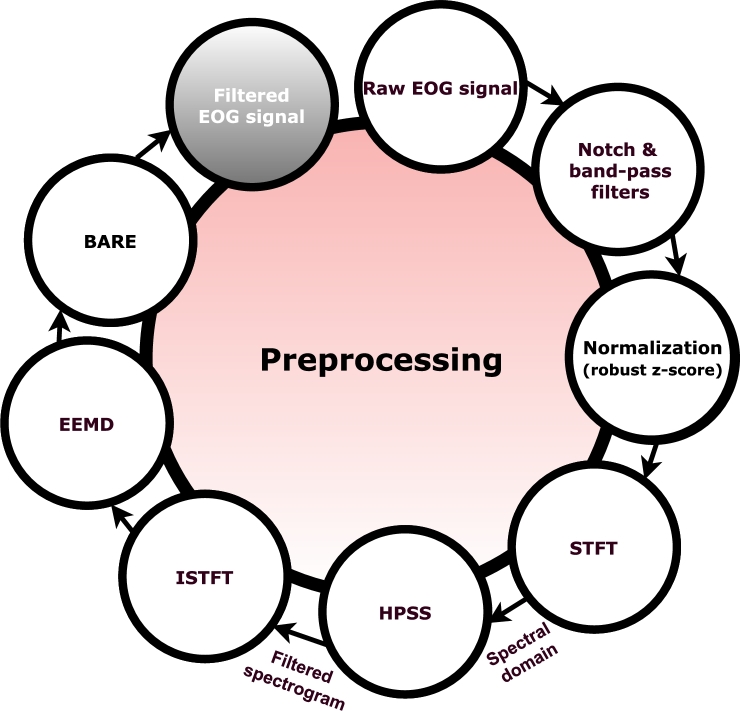
Complete preprocessing protocol.

Fig. 6 depicts the diagram of the noise reduction process. Within the scope of this paper, the term *without harmonics enhancement protocol (WHE)* is attributed to the approach employing steps one to four of the preprocessing protocol plus BARE, for removing the eye-blink artifacts except additional HPSS and EEMD processes. The other two exploratory analyses include all the steps in the *HPSS protocol* and *HPSS + EEMD protocol*, respectively.

The source code of the harmonic enhancement protocol alongside with BARE function can be reached through this link: github link.

### 2.8 Feature extraction

The preprocessed continuous EOG signals are divided into one-second windows with 500 ms padding. A 36-dimensional feature set spanning multiple analytical domains is chosen to capture the intricate dynamics of EOG signals, incorporating temporal, spectral, entropy-based, amplitude, area-under-curve, statistical, variability, wavelet, and complexity features. This selection is designed to provide a comprehensive representation by analyzing multifaceted signal characteristics, frequency distributions, predictability levels, signal magnitude, integrated signal strength, distribution properties, signal variability, time-frequency details, and the signal's inherent complexity. The set begins with three spectral features: centroid [46], bandwidth (SB) [47], and roll-off [48], focusing on the signal's frequency domain. It then includes sixteen temporal features: zero-crossing rate (ZCR) [49], short-term energy [49], short-term energy entropy [49], polynomial features [50], wavelet decomposition [51], variance and mean of vertex to vertex slope, and root-mean-square value. Additionally, ten dimensions are dedicated to amplitude and statistical features: maximum peak amplitude (PAV) [52], minimum valley amplitude (VAV) [52], the area under the curve (AUC) [52], variance, and statistical descriptors including kurtosis, skewness, mean, median, standard deviation and the coefficient of variation. The set is rounded off with seven entropy and complexity features, comprising various entropy calculations like permutation entropy (PE) [53], spectral entropy (SE) [49], [54], approximate entropy (ApEn) [55], and sample entropy (SampEn) [55], as well as three variations of Lempel-Ziv complexity (LZc) [56] measures. This structured approach ensures a multidimensional understanding of the EOG signal, encapsulating its dynamic, spectral, and complex nature. The features used in this study are:

• ZCR: the rate of sign-changes along signal. ZCR formula is shown in Eq. (6).
  (6) $$ZCR=\frac{1}{T-1}\sum_{t=1}^{T-1}1_{R<0}(S_{t},S_{t-1})$$
  where $S_{t}$ is a signal point at time *t*, and the signal has a length of *T*. R<0 is an indicator function to select only the signal's time points that change their amplitude from positive to negative.
• Short-Term Energy: the sum of squares of the samples in the given window, with its formula shown in Eq. (7).
  (7) $$E_{T}=\frac{\sum_{m=0}^{N-1}s^{2}(m)}{N}$$
  where *N* is the total size of the discrete window and s(m) is the energy of the given time point at *m*.
• Short-Term Energy Entropy: it is the entropy of averaged sub-frame energies, shown in Eq. (8).
  (8) $$E_{\text{tot}}=\sum_{m=0}^{N}S{\left(m\right)}^{2}\;E_{\text{sub}}=\sum_{b=0}^{N÷K}\sum_{a=0}^{K}S{\left(a\right)}^{2}\;E_{\text{entropy}}=-\sum \frac{\sum E_{\text{sub}}^{2}}{E_{\text{tot}}}\log\left(\frac{\sum E_{\text{sub}}^{2}}{E_{\text{tot}}}\right)$$
• Spectral Centroid: a metric for characterizing a spectrum. It indicates the location of the center of mass of the spectrum. The formula of the Spectral Centroid is shown in Eq. (9).
  (9) $$C=\frac{\sum_{n}^{N-1}f(n)x(n)}{\sum_{n=0}^{N-1}x(n)}$$
  where x(n) represents the weighted frequency value and f(n) represents the center frequency of a bin.
• Spectral Bandwidth: formula of p'th order Spectral Bandwidth is shown in Eq. (10).
  (10) $$\mathrm{SB}=\sum_{K}S(k)\;{\left(f(k)-{\mathrm{centroid}}^{p}\right)}^{\frac{1}{p}}$$
  where S(k) represents the energy bin of the spectrum, f(k) represents the center frequency of that bin, and *p* is the order of spectral bandwidth.
• Spectral Roll-Off: the roll-off frequency is defined as the central frequency of a spectrogram bin that contains at least 0.85% (by default) of the frame's energy.
• Polynomial Features: it mainly provides the k-th order polynomial fitting of the frequency spectrum of a given signal. Coefficients of found polynomials are accepted as extracted features.
• PAV: measures the peak signal amplitude.
• VAV: measures the lowest negative amplitude value.
• AUC: the total energy of positive and negative absolute amplitude values.
• Statistical features: there are five fundamental statistical outcomes of a time-serie including kurtosis, skewness, mean, median and STD.
• Coefficient of variation: a statistical metric that is frequently used to calculate the diversity of a series by taking the ratio of the STD to the mean.
• Wavelet decomposition: the examination of localized fluctuations in power within a time series uses wavelet analysis. It can be defined as a discrete wavelet transformation (DWT) to reconstruct the waveform via convolving with low and high-pass filters. Instead of taking the whole filtered signal as a feature, statistical time-series representations (mean, STD, some of the squared amplitudes, and entropy value) are extracted from the transformed signal as an additional feature space.
• LZc: a metric for detecting dynamic changes in non-linear signals, which measures the number of new patterns in the time series. Before computing the LZc measure, the signal must be transformed into a binary sequence by comparison with the threshold. In this study, three distinct varieties of LZc are utilized, each determined by different threshold values for binarization, where values above the threshold are set to 1, and those below the threshold to 0. The first variety (Feature-1) is calculated using the median value of the signal, the second variety (Feature-2) employs the mean value of the Hilbert-transformed series, and the third variety (Feature-3) is based on the mean value plus 1.3 times the standard deviation (*std*) of the signal. [57] gives an extensive description of the LZc algorithm.
• Entropy
  **–** PE: A natural complexity measure for time series based on comparison of neighbouring values. The PE for order n≥2 is formalized in Eq. (11):
    (11) H(n)=−∑p(π)logp(π)
    where *n* is the number of dimensions.
  **–** SE: the entropy of the power spectrum represents the relative flatness or peakedness of the spectral distribution. Thus, it can be considered a measure of signal irregularity degree. Spectral entropy over the whole frequency range is formalized in Eq. (12):
    (12) $$H=-\sum_{f=0}^{f_{n}}S(f)log_{2}S(f)$$
    where the upper limit frequency, $f_{n}$ is selected as high within the cutoff frequency and the units of *H* are bits.
  **–** ApEn: a quantification of the rate of regularity in time data series. Low ApEn values reflect a persistent and predictive system, whereas high values point to a low number of repeated patterns and randomness. Originally it was developed to handle the limitations of exact regularity statistics, which require vast amounts of data and are greatly influenced by system noise.
  **–** SE: a modification of the ApEn, which brings two advantages: data length independence and relatively trouble-free implementation.

### 2.9 Feature set normalization

The feature set is z-score normalized as shown in Eq. (13).

(13) $$\mathrm{Normalized}(s_{i})=\frac{s_{i}-\mu (S_{i})}{\sigma (S_{i})}$$

where $S_{i}$ is feature sequence of *features*_*i*_, and $s_{i}$ is one sample that will be normalized.

### 2.10 Recursive feature elimination

The redundant features are removed from the entire space using recursive feature elimination with 10-fold cross-validation (RFECV) [58]. Due to its suitability for deriving feature significance coefficients for each feature, the decision tree classifier [59] is employed.

### 2.11 Hyperparameter optimization and selection

For all classifiers, the optimum hyperparameters are determined in the initial step using hyperparameter optimization. Alternative optimization techniques are designed to identify a relatively decent hyperparameter combination rather than a grid search. The latter attempts all hyperparameter combinations to find the best one, resulting in a very high computation cost. Instead, Bayesian hyperparameter optimization [60] is used to find the model parameters. The advantage of this method is that it is computationally cheaper than brute force methods and converges quickly to high classification performance.

### 2.12 Feature engineering

Incorporating a time-domain-dependent semantic context, the strategy of combining features from the consecutive overlapping windows in the realm of the subject-dependent analyses, overlapping & sliding windowing enhances the classification performance of activity decoder models [61]. This approach, rooted deeply in the principles of time-series analysis, leverages the intrinsic semantic relationships between adjacent time intervals to augment the feature substantially. Overlapping windows, especially short-time ones, are adept at capturing consistent patterns of muscle activity, which are pivotal in delineating distinct activities. Such a method ensures that the temporal continuity and the subtle transitions between these movements are accurately represented, enriching the dataset with nuanced features otherwise overlooked in non-overlapping schemes. This practice of feature space expansion through overlapping windows, which effectively increases the total dimensionality, is not only well-established but also widely validated in the realm of human activity recognition [62][63][64][65]. Accordingly, the features of the three consecutive windows are combined to create an expanded feature set with a total dimensionality of 108 (3⁎36). It allows for a more detailed and comprehensive capture of the dynamic characteristics of EOG signal patterns, thereby facilitating a more nuanced analysis and improved prediction performance. Selection of three consecutive windows strikes a balance between maintaining high temporal resolution and ensuring continuity in the feature space. It allows for capturing transient dynamics within the EOG signal that may be missed with wider windows while avoiding the redundancy and computational load of using more windows.

### 2.13 Dual input deep learning architecture

In this study, a dual input deep learning model is developed to enhance the classification performance of ocular activity decoding. The model combines the time-frequency (TF) representation of preprocessed EOG time-series using Morlet wavelets [66] with the vectorized feature set, fed through bidirectional long-short term memory (LSTM) [67]. The TF representation, obtained via Morlet wavelets, captures the spectral information of the EOG signals, while the vectorized feature set captures the temporal characteristics. This combination allows for a comprehensive representation of the ocular activities.

A two-dimensional feature input is constructed via vertical stacking of three adjacent feature sequences to use a Bidirectional LSTM and incorporate the time dimension as an additional factor. The vectorization process is applied to facilitate this transformation.

The constructed TF maps are decimated from the resolution of (500x501x3(time)) to (20x26x3), allowing to reduce substantially the learning process. Bayesian hyperparameter optimization is also employed in this architecture to optimize the fixed amounts of convolutional, LSTM, and dense layers (two of each). The parameters tuned included the number of units in the first and second LSTM layers, which are varied between 50 and 300 units. Similarly, the number of filters in the first and second CNN layers are selected from the options of 32 and 64 filters. Additionally, the number of neurons in the first and second dense layers is adjusted within the range of 50 to 200 neurons. Consequently, the final model includes 100 neurons (50⁎2 for forward-backward processing). A dropout rate of 0.45 is applied after each LSTM layer, and in the final step, flattening is performed to extract the feature set of 300 dimensions.

On the other side, a 2D Convolutional Neural Network (CNN) [68] is constructed with both the first and second convolutional layers with 32 filters (2x2), followed by leaky ReLU activation with an alpha value of 0.1. Next, a max-pooling layer of 2x2 is applied, followed by Batch Normalization. A dropout rate of 0.35 is applied after Batch Normalization, and the final flattening operation extracts a 620-dimensional feature set.

**Figure 7:**
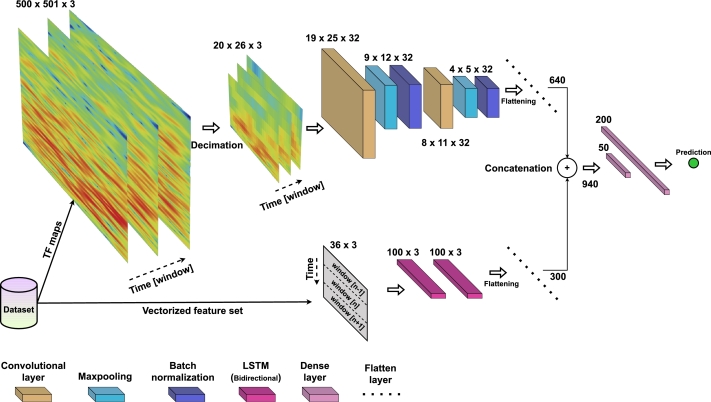
Dual input learner model.

In the final step of the model, both flattened layers are concatenated, and a 940-dimensional feature set is fed into two-layer dense networks with 50 and 200 neurons, respectively. The activation function used is leaky ReLU [69], and a dropout rate of 0.4 is applied after each activation. The final dense layer connects to the softmax layer. Adamax [70] with a learning rate of 0.005 is the chosen optimizer. The optimum parameters are found based on 150 iterations of Bayesian optimization. Fig. 7 depicts the final model architecture.

## 3 Analysis and results

### 3.1 Classifier selection

A total of thirteen different classifiers are chosen: dual input learner model, linear discriminant analysis (LDA) with shrinkage value, K-nearest neighbors (KNN), Gaussian process classifier (GPC) [71] based on Laplace approximation, Bagging classifier [72], support vector machine classifier (SVC) [73], XGBoost [74], Random forest, Lightgbm (LGBM) [75], Voting ensemble classifier [76], stochastic gradient descent (SGD) [77], stack ensemble meta-model [78] and the standalone CNN model, which uses only the spectrogram image as input, is utilized as a comparative model alongside the dual-input learning model. The stack ensemble uses stacking as a tool for meta-model training by combining the results from independently trained classifiers (KNN, GPC, Bagging, Adaboost, SVC, SGD, XGBoost, Random Forest, and LGBM). It uses the stochasticity of the predictions from each model, and the meta-model makes a final decision from incoming inputs. A voting classifier with a soft voting mechanism predicts the argmax of total probabilities. Sklearn [79] and Keras [80] packages are chosen to execute the algorithms in the Python3.9 environment.

#### 3.1.1 Leave-one-session-out cross validation results

The full dataset is divided into nine folds according to leave-one-session-out cross-validation (LOSO) [81]. Correspondingly, n−1 out of *n* subject data is trained, and the remaining subject data is used as a test. This approach prevents data leakage from the test subject into the training dataset, thus producing performance results that are substantially more generalizable than traditional k-fold cross-validation with shuffling. Within each fold, the training and test data (test subject) are normalized according to the mean and standard deviation data of the training set individually for each feature. The subsequent step involves applying RFECV within each LOSO training subset to identify the most pertinent features from the 108-dimensional feature set. On average, across all LOSO sub-training datasets, the following features are selected for their relevance, listed in decreasing order of selection frequency: spectral band, skewness, mean, SE, short-term energy entropy, vertex & vertex slope, PE, ZCR, kurtosis, LZC Hilbert, ApEn, polynomial features, wavelet decomposition, VAV, LZC median, SE, PAV, LZC mean, spectral centroid, coefficient of variation, AUC, spectral roll-off, variance, median, standard deviation, short-term energy, and RMS. After this process, only the training set is subjected to hyperparameter optimization, and the test data are isolated from this process.

The LOSO performances of each model with and without harmonic filtering of EOG signals are shown in Table 1. The evaluation metrics include mean accuracy (Acc.) with standard deviation based on individual fold performances, precision (Prec.), recall (Rec.), and f1-score (F1-sc.). Given that the sample sizes of the classes are equal, the accuracy metric is more decisive than the others and chosen accordingly. HPSS + EEMD filtering outperforms other filtering protocols (WHE and HPSS) in every classifier. A dual input learner provides the highest accuracy (72.6%) with the HPSS and EEMD hybrid approach. Stack ensemble model close behind with 71.2%. CNN, SVC, LGBM, XGB, Voting soft, random forest, GPC, LDA, KNN, Bagging, and SGD models give the overall accuracy of 70.6%, 70.4%, 70.1%, 69.9%, 69.9%, 68.6%, 67.8%, 67.7%, 66.7%, 66.3% and 65.5% respectively.

**Table 1:** LOSO classification results across filtering protocols.

Model	WHE				HPSS				HPSS + EEMD			
	Acc.	Prec.	Rec.	F1-sc.	Acc.	Prec.	Rec.	F1-sc.	Acc.	Prec.	Rec.	F1-sc.
***Dual input learner***	**0.688** ± **0.074**	**0.714**	**0.688**	**0.664**	**0.713** ± **0.099**	**0.736**	**0.713**	**0.687**	**0.726** ± **0.074**	**0.750**	**0.726**	**0.702**
*Stack ensemble*	0.671 ± 0.071	0.690	0.671	0.650	0.689 ± 0.087	0.712	0.689	0.668	0.712 ± 0.062	0.737	0.712	0.691
*CNN*	0.667 ± 0.068	0.687	0.668	0.644	0.685 ± 0.096	0.710	0.686	0.666	0.706 ± 0.089	0.731	0.706	0.684
*SVC*	0.665 ± 0.074	0.687	0.665	0.643	0.683 ± 0.083	0.709	0.683	0.662	0.704 ± 0.071	0.727	0.704	0.681
*LGBM*	0.669 ± 0.064	0.687	0.669	0.644	0.693 ± 0.078	0.727	0.693	0.667	0.701 ± 0.061	0.727	0.701	0.676
*XGB*	0.663 ± 0.067	0.678	0.663	0.639	0.686 ± 0.081	0.716	0.686	0.659	0.699 ± 0.063	0.723	0.699	0.674
*Voting soft*	0.672 ± 0.081	0.700	0.672	0.650	0.695 ± 0.089	0.727	0.695	0.671	0.699 ± 0.065	0.731	0.699	0.675
*Random forest*	0.657 ± 0.062	0.677	0.657	0.627	0.680 ± 0.076	0.714	0.680	0.651	0.686 ± 0.060	0.725	0.686	0.656
*GPC*	0.644 ± 0.076	0.682	0.641	0.622	0.669 ± 0.094	0.714	0.669	0.647	0.678 ± 0.069	0.724	0.678	0.657
*LDA*	0.630 ± 0.116	0.681	0.630	0.609	0.658 ± 0.133	0.727	658	0.644	0.677 ± 0.104	0.732	0.677	0.663
*KNN*	0.637 ± 0.077	0.686	0.637	0.619	0.663 ± 0.091	0.710	0.663	0.644	0.667 ± 0.066	0.716	0.667	0.649
*Bagging*	0.632 ± 0.067	0.658	0.632	0.605	0.671 ± 0.077	0.696	0.671	0.646	0.663 ± 0.067	0.701	0.663	0.634
*SGD*	0.627 ± 0.087	0.651	0.627	0.590	0.662 ± 0.083	0.699	0.662	0.642	0.655 ± 0.068	0.692	0.655	0.628

In addition, the normalized confusion matrix of the dual input learning model (the highest performing model) LOSO scores are extracted and shown in Fig. 9 by rounding off values to two decimal places. Accordingly, eye blink activity is undoubtedly the best-predicted class (94.52%). This performance is followed by right and left eye closed activities (86.29%, and 85.43%). Normal glance and eyebrows up yielded considerably lower classification results (71.28%, and 53.10%). Frowning achieved the poorest classification success with a performance of 45.45%.

### 3.2 Evaluation of LOSO performances

**Figure 8:**
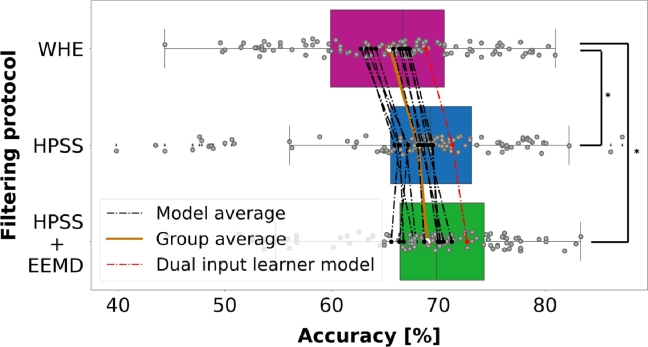
Accuracy comparison across LOSO performances of filtering protocols. The star (*) on the right side of the figure denotes statistically significant differences between the conditions.

Classification performance across the three preprocessing protocols (WHE, HPSS only, and HPSS with EEMD) is statistically compared via one-way repeated measures ANOVA (RMA) within-subject comparison. Accuracy is chosen as a corresponding metric. All LOSO scores for the three filtering scenarios are chosen as a pair. Prior to conducting the RMA, Mauchly's test of sphericity was employed to assess the equality of variances among the pairs in the within-subject condition. The results indicated unequal variances, necessitating the Greenhouse-Geisser (GG) correction on the significant RMA values. The RMA analysis revealed statistically significant differences in the LOSO performances across all filtering types, as presented in Table 2. A further post hoc test is applied to highlight the performance differences of each protocol. Holm correction test is chosen due to having a relatively high number of LOSO values in each group, providing a more robust evaluation. Accordingly, the LOSO scores of WHE are significantly lower than that of the other groups (WHE vs. HPSS: **p < .001** and WHE vs. HPSS + EEMD: **p < .001**). However, no statistically significant difference is observed between HPSS and HPSS & EEMD combination considering all model performances (**p = .065**). Nevertheless, the grand average of LOSO accuracy for all classifiers increased by 0.89% when combining HPSS and EEMD, as compared to HPSS alone. Additionally, the dual input learner model exhibited an approximately 1.3% decoding improvement after incorporating both HPSS and EEMD as opposed to HPSS alone. The performance comparisons are visually depicted in Fig. 8.

**Table 2:** RMA within-subject comparisons of accuracies across the filtering types.

**Table:** **RMA** SS df MS F $P_{GG\;\;\mathrm{Corrected}}$ 0.070 1.806 29.244 8.007 p = <.001
**Table:** 95% CI for mean difference Group 1 Group 2 Mean dif. Lower Upper SE t *P*_*Holm*_ WHE HPSS -0.026 -0.037 -0.015 0.005 -5.498 p < .001 WHE HPSS + EEMD -0.035 -0.046 -0.023 0.005 -7.353 p < .001 HPSS HPSS + EEMD -0.009 -0.020 0.003 0.005 -1.855 p = .065

**Figure 9:**
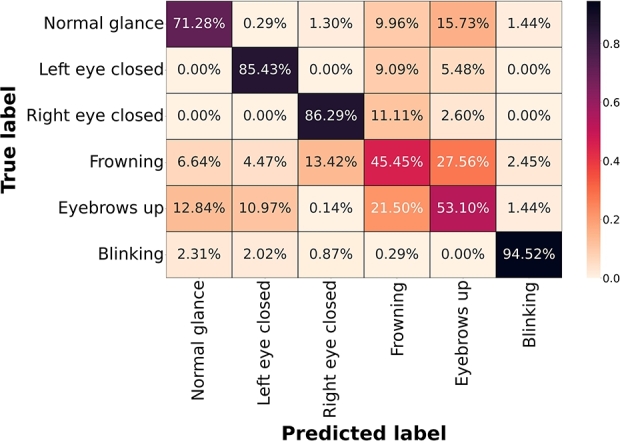
Normalized Confusion Matrix of the overall LOSO performance of the dual input learner model.

To provide a comparative analysis with the literature, the present research applies 5-fold cross-validation with shuffling to evaluate the performance of the investigated filtering protocols using the dual input learner model. The WHE protocol achieves an accuracy of 79.2% ± 1.58%, with the f1-score, precision, and recall scores of 78.7%, 78.8%, and 79.2%, respectively. The HPSS protocol demonstrates an accuracy of 84.4% ± 1.37%, along with f1-score, precision, and recall scores of 84.3%, 84.6%, and 84.4%, respectively. Notably, the HPSS + EEMD combination exhibits the highest performance across all protocols, achieving an accuracy of 87.2% ± 1.8%, along with f1-score, precision, and recall scores of 87.3%, 88.1%, and 87.2%, respectively. The results of the relevant literature studies employing the same evaluation metrics are briefly presented in Table 3.

**Table 3:** Comparison of results with some existing literature. Minimal-sensing refers to a single-channel configuration and a reference channel, while multi-sensors represent an EOG setup with more than one channel other than a reference.

Study	[82]	[83]	[84]	The proposed study
#classes	6	5	3	6
#sensors	minimal-sensing	multi-sensors	minimal-sensing	minimal-sensing
#subjects	10	12	8	9
Evaluation method	Train & test	Cross-validation	Train & test	Cross-validation
	split for each subject		split for each subject	
	(online classification)			
Accuracy	85%	76.9%	84%	87.2%

### 3.3 Correlation between harmonic ratio and prediction performances of static activities

Based on the confusion matrix, it can be inferred that there are varying performance levels in the different activities. Particularly for frowning and eyebrows-up activities, the performance is poor. To understand this discrepancy the harmonic powers of static activities, excluding blinking activity, are compared to the HHT-PSD spectrum. The fundamental frequency (F0) and its exact multiples (1st harmonic, 2nd harmonic, and 3rd harmonic) are visually determined. Further, to numerically describe the intensity of harmonic components of a spectrum, the harmonic ratio is calculated. This calculation involves dividing the sum of energy values at harmonic frequencies by the energy of the entire spectrum, with the resulting ratios depicted in Eq. (14).

(14) $$HP(i)=\sum_{F}\sum_{f=f_{harm\;min}}^{f_{harm\;max}}PSD_{i}(f)HR(i)=\frac{HP(i)}{max(HP)}$$

where *HP* is a harmonic power and *HR* is a harmonic ratio of *PSD* extracted via HHT by dividing each activity power by the maximum one (max(HR)). *i* specifies the activity, *F* represents the collection of harmonic frequency thresholds exemplified as $f_{harmmin}$ and $f_{harmmax}$.

**Figure 10:**
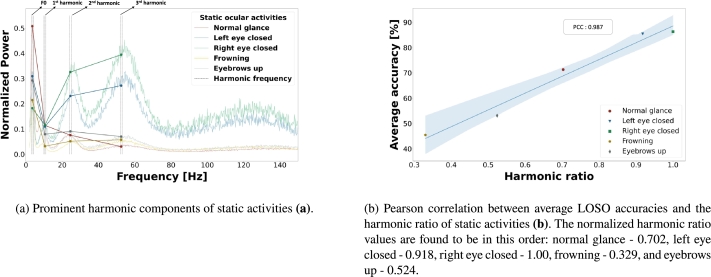
Harmonic components of static ocular activities and the association between harmonic ratios and model performances. The prominence of harmonic peaks in higher-performing activities can be seen in **(a)**. Determined harmonic components are further assessed by calculating the harmonic ratio, and the ratio values of all static activities are correlated with the averaged LOSO performance of the dual input learner model **(b)**. The harmonic ratio values are normalized between [0-1].

Subsequently, the harmonic ratio is extracted for all static activities, and a Pearson correlation (PCC) analysis is conducted to correlate it with the average performance values. Accordingly, a PCC value of 0.987 indicates that the prominence of harmonic components in activities correlates with the distinct features that differentiate it from others, thereby influencing the chosen model's (dual input learner) performance. The variation of harmonic intensity across conditions (Fig. 10a) and the effectiveness of the harmonic ratio to the averaged validation performance of the dual learner model (Fig. 10b) are illustrated.

## 4 Discussion

This study examines the effectiveness of the harmonic components of EOG-based ocular activities for decoding. Instead of preserving only the low-frequency range remarkably high frequencies are retained, and harmonic amplification is employed by suppressing the percussive components using the HPSS filter. The combination of EEMD with the HPSS filter provides an enhancement in classification performance.

During the EEMD process, each extracted IMF inherently captures distinct oscillatory components, predominantly those within the harmonic spectrum. The effectiveness of EEMD in separating these components is attributed to the incorporation of white noise in the decomposition process. A vital aspect of this method comprises aggregating individually oriented frequency components from separate IMFs. This merging process seemingly enhances the clarity and, putatively, increases the detectability of the F0 and its harmonics. The summation of the IMFs results in harmonic enhancement, which enriches the harmonic representation of the signal and increases the decoding performance of the ocular activities.

Given that the subjects perform eye movements as they would in a naturalistic environment, the entire dataset with static activities is contaminated with reflexive and spontaneous blinks. The novel BARE algorithm created to replicate the probable true signals instead of blinking peak artifacts allows cleaning these artifacts from the consecutive positive-negative patterns by minimizing the information loss. It considers the interquartile range of both sides around a detected peak and replaces the region with the signal's envelope. This approach allows retaining the continuation, thus shrinking the computation time by focusing solely on blink regions.

After feature extraction, RFECV is applied before training most machine learning models, but not in our custom dual input learner-based deep learning model. This decision is based on the LSTM component's (one of the sub-branches of the dual input learner model) ability to capture complex, non-linear data relationships [85]. Employing feature reduction like RFECV could result in significant information loss. LSTMs excel in processing and learning from raw, high-dimensional sequential data, autonomously identifying key features. By avoiding feature reduction, we ensure the LSTM accesses the full spectrum of data, preserving its capacity for uncovering intricate patterns and temporal dependencies. This approach optimizes the LSTM's functionality and enhances overall performance in our deep learning model. The performance of each model is evaluated using the LOSO method on the validation dataset, considering four metrics. Next, the LOSO performances of the three preprocessing scenarios are compared via RMA in a within-subject analysis for the accuracy metric. The HPSS filter significantly improves the signal quality of EOG activities compared to WHE preprocessing. However, the addition of EEMD to HPSS does not provide a statistically significant boost to decoding performance. Nonetheless, the average accuracy of all models increases by 0.89%, and the dual input learner model's accuracy, which yields the best performance in all scenarios, is boosted by 1.3%. The most prominent performance boost is 3.8% comparing the baseline WHE condition with HPSS and EEMD combined protocol. These findings support our assumption that preserving and enhancing the harmonic information, including very high frequencies, is beneficial for signal decoding.

The confusion matrix of the dual input learning model indicates remarkable variations in individual activity performance. Activities such as frowning and raising eyebrows exhibit relatively poorer performance compared to normal glance, left and right eyes closed, and blinking. The deviations in performance gaps are further investigated by analyzing the properties of harmonic components. The harmonic ratio of each static ocular activity is correlated with individual activity decoding performance. A high correlation between the harmonic ratio and the overall accuracies of static ocular activities indicates that activities with higher distinctiveness in harmonic properties yield superior decoding performance. This finding offers a new perspective for exploring EOG activities and could potentially enhance the decoding of other biopotential sensor activities by defining and understanding their harmonic structures and intensities. Accordingly, the studies in various fields, such as defining musical genres via harmonic structures [86], determining fatigue through speech signals using the harmonic-to-noise ratio (HNR) [87], and diagnosing machine failures through vibration analysis based on harmonic properties [88], coincide with this perspective.

Despite the definition of only three harmonics alongside the F0 defined in the PSDs extracted via HHT (Fig. 10), it is noteworthy that the high-frequency components are considered non-arbitrary. The employment of the broad frequency spectrum boosts the decoding performance, suggesting the possibility of additional harmonic-like structures in the high-frequency range. Additionally, rather than traditional peak-to-peak components, a widened structure may represent a harmonic component, as observed in the PSDs of left and right eye closed activities (between 100 and 150 Hz).

It is crucial to consider the characteristics and limitations of each study when interpreting the results. Direct comparisons may not bring insights due to substantial differences in the evaluation methodologies, apart from the multi-sensory streaming in some cases. It is noteworthy that the reference studies mentioned either apply cross-validation with shuffling or utilize adaptive models for each subject. In this regard, cross-validation is also applied in the proposed study to compare with the literature, and the overall accuracy of the EOG activity decoding performance in the hybrid approach is 87.2% - the highest across similar studies.

Given that a minimal sensing approach is adopted in the current study, this strategy is designed to balance the need for simplicity and efficiency in data acquisition while striving to preserve the distinct characteristics inherent to various EOG activities. The main motivation is to ensure comparability with other minimal sensing studies and to underscore the effectiveness of the proposed harmonic enhancement. Inherently, this approach limits the spatial resolution of sensory data, particularly impacting the detection of vertically oriented ocular activities such as frowning and eyebrows-up. These activities have lower decoding performance due to a significant reduction in harmonic intensity (Fig. 10), in contrast to left and right eye-closed activities. The current EOG design's limitation in capturing ocular movements oriented along the vertical plane affects the algorithms' decoding accuracy for such activities. Enhancing the setup with additional channels, specially aligned to detect vertical ocular movement, could enhance the decoding performance by expanding the sensory array. For instance, one could have a setup resembling that of [89], yet preserving higher frequency components and employing our hybrid approach for event decoding.

The balance between the simplicity of the sensor setup and the complexity of ocular movements underscores the importance of continuously evaluating acquisition strategies in EOG-based activity analysis. Future studies should aim for multi-channel EOG placements incorporating both horizontal and vertical electrodes, allowing for a more comprehensive exploration of ocular activity variations (ensuring nuanced sensitivity to a full range of ocular activity directions) and their impact on harmonic components by preserving very high frequencies (ideally until Nyquist frequency). Such enhancements are anticipated to increase detection sensitivity, particularly for activities characterized by pronounced vertical movement. This improvement comes from integrating various harmonic spectra (namely, through regularization-based averaging across EOG harmonic spectra to enhance harmonic intensity), aiming to achieve greater overall harmonic intensity. Different combinations of activities will be evaluated individually, prioritizing those with distinct harmonic characteristics to enhance decoding capabilities for a broader range of ocular activities (extreme classification systems while minimizing the loss of evaluation performance). These advancements hold promise for expanding the potential applications and possibilities in the field of HMI.

## 5 Conclusion

In conclusion, a hybrid preprocessing protocol is developed to enhance prediction performance in machine learning-based systems for EOG-based static activity signals by amplifying harmonic components, including high frequencies. Comparative analysis of WHE preprocessing, HPSS, and a hybrid HPSS-EEMD approach is conducted. Feature sets are extracted and LOSO performances are evaluated with various machine learning models. Results demonstrate that HPSS significantly outperforms WHE by emphasizing harmonic components. While adding EEMD to HPSS does not provide a statistically significant improvement, it results in an average performance increase of 0.89% and 1.3% for the dual input learner model. The highest performance is achieved by a model using dual input: vectorized feature sets and TF maps. Overall, the hybrid harmonic enhancement approach boosts performance by 3.8% compared to the WHE protocol. A correlation is established between prediction performance and harmonic ratios, indicating that activities with higher harmonic properties have greater discriminability and higher prediction performance. The current study is limited by the minimal sensing EOG recording, which lacks comprehensive spatial sensitivity. Future research could address this limitation by utilizing multi-channel EOG placements spanning both vertical and horizontal domains around the ocular muscles, improving decoding accuracy of a broader range of ocular activities by preserving high-frequency components near the Nyquist frequency, potentially enhancing sensitivity for various HMI applications.

## Ethics statement

Each subject provided written informed consent prior to participation. Approval by an ethics committee was not required for this study in accordance with the local legislation and institutional requirements, given its non-clinical, non-invasive nature. Moreover, the characteristics of the recorded data safeguard participant privacy by preventing the identification of personal details. This careful consideration of participant comfort, the simplicity of the procedure, and the protection of privacy adheres to ethical guidelines for minimal-risk studies, ensuring the well-being of all involved without compromising anonymity.

## CRediT authorship contribution statement

**Çağatay Demirel:** Writing – original draft, Visualization, Methodology, Formal analysis, Data curation, Conceptualization. **Livia Reguş:** Writing – review & editing, Visualization. **Hatice Köse:** Supervision.

## Declaration of Competing Interest

The authors declare that they have no known competing financial interests or personal relationships that could have appeared to influence the work reported in this paper.

## Data Availability

Data will be made available on request
